# Synthesis, characterization, and water oxidation by a molecular chromophore-catalyst assembly prepared by atomic layer deposition. The “mummy” strategy†
†Electronic supplementary information (ESI) available: Synthetic descriptions, cyclic voltammograms, absorption spectra, photocurrent traces. See DOI: 10.1039/c5sc01752a


**DOI:** 10.1039/c5sc01752a

**Published:** 2015-07-31

**Authors:** A. M. Lapides, B. D. Sherman, M. K. Brennaman, C. J. Dares, K. R. Skinner, J. L. Templeton, T. J. Meyer

**Affiliations:** a Department of Chemistry , University of North Carolina at Chapel Hill , CB 3290 , Chapel Hill , NC 27599 , USA . Email: joetemp@unc.edu ; Email: tjmeyer@unc.edu

## Abstract

A new strategy for preparing spatially-controlled, multi-component films consisting of molecular light absorbing chromophores and water oxidation catalysts on high surface area, mesoporous metal oxide surfaces is described. Atomic layer deposition (ALD) is used to embed a surface-bound chromophore in a thin layer of inert Al_2_O_3_, followed by catalyst binding to the new oxide surface. In a final step, catalyst surface-binding is stabilized by a subsequent ALD overlayer of Al_2_O_3_. The ALD assembly procedure bypasses synthetic difficulties arising from the preparation of phosphonic acid derivatized, covalently-linked assemblies. An ALD mummy-based assembly has been used to demonstrate photoelectrochemical dehydrogenation of hydroquinone. Electrocatalytic water oxidation at pH 8.8 is observed over a 2 hour electrolysis period and light-assisted water oxidation over a 6 hour photolysis period with O_2_ detected with a generator–collector electrode configuration.

## Abstract 2

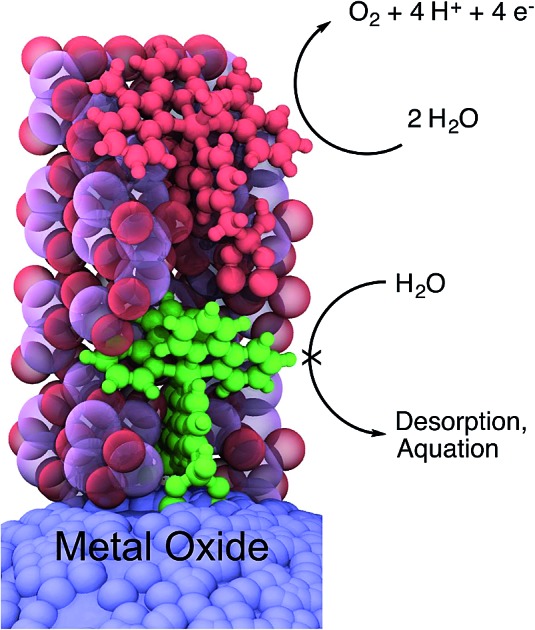
A Ru(ii)-polypyridyl chromophore-catalyst assembly for light-assisted water oxidation is constructed using atomic layer deposition with no covalent bonds between molecules required for bilayer formation.

## Introduction

In a Dye-Sensitized Photoelectrosynthesis Cell (DSPEC) for water splitting, a molecular light absorber and catalyst are integrated with a wide bandgap metal oxide semiconductor.^1^–^3^ Typically, the molecular components are either surface-bound on the oxide or covalently linked prior to surface attachment with phosphonate-surface binding used for aqueous stability.^4^–^6^ A number of alternate assembly strategies have been explored including a layer-by-layer technique,^7^,^8^ electro-assembly formation,^9^–^11^ and pre-formed polymer and peptide assemblies.^12^

Although reasonably stable in acidic solutions, phosphonate surface binding is unstable toward hydrolysis as the pH is increased above 5.^13^ An additional stability issue arises from decomposition of the oxidized forms of most chromophores under aqueous conditions which also limits DSPEC stability and performance over extended periods.^14^–^18^ Atomic layer deposition (ALD), with thin overlayers of aluminum oxide (Al_2_O_3_ or AO) or titanium dioxide (TiO_2_) added after surface binding, has been used successfully to stabilize phosphonate-surface binding even at high pH.^19^–^22^ We describe here a new ALD-based “mummy” strategy for preparing and stabilizing chromophore-catalyst assemblies. It utilizes ALD for both forming and stabilizing assemblies without the need for covalent or ionic bonds between units. The assembly process is stepwise involving: (1) initial surface binding of a chromophore; (2) embedding the chromophore in a thin layer of deposited oxide; (3) surface binding of a molecular catalyst; and, finally, (4) thin-layer deposition of an oxide overlayer to stabilize surface binding of the catalyst. Here we describe the application of this strategy to the preparation of a Ru(ii) polypyridyl chromophore-catalyst assembly on nanoparticle films of two oxides, tin-doped indium oxide (*nano*ITO) for electrocatalytic water oxidation and titanium dioxide (*nano*TiO_2_) for light-assisted photoelectrochemical water oxidation.

## Results and discussion

### Bilayer formation

**Fig. 1:**
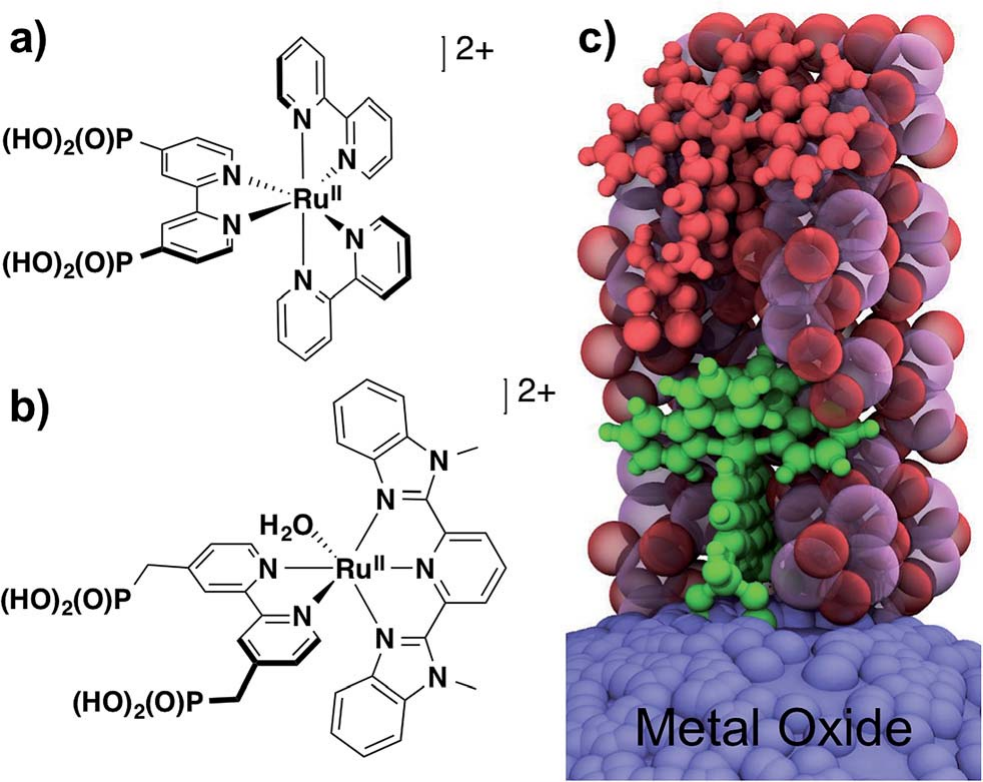
Molecular structures of: (a) chromophore, **RuP^2+^**; and (b) water oxidation catalyst, **RuCP(OH_2_)^2+^**. (c) Visualization of the ALD mummy protected surface assembly with **–RuP^2+^** (green molecule) and **–RuCP(OH_2_)^2+^** (red molecule) embedded in ∼3 nm of Al_2_O_3_.

The chromophore [Ru^II^(4,4′-((HO)_2_(O)P)_2_-2,2′-bipyridine)(2,2′-bipyridine)_2_]^2+^ (**RuP^2+^**, Fig. 1a) was synthesized as its chloride salt as previously described.^23^ Films of *nano*TiO_2_ and *nano*ITO were loaded with **RuP^2+^** by soaking in methanol solutions (∼1 mM in complex) overnight to give *nano*TiO_2_**|–RuP^2+^** or *nano*ITO**|–RuP^2+^**. Surface coverages were determined by UV-visible absorption measurements with *ε* = 12 700 M^–1^ cm^–1^ at *λ*_max_ = 458 nm for a solution analog.^9^

ALD overlayers of aluminum oxide (Al_2_O_3_; AO) were deposited atop derivatized *nano*ITO**|–RuP^2+^** electrodes by sequential pulses of Al(CH_3_)_3_ and H_2_O at 150 °C under dynamic vacuum. Ellipsometry performed on a witness Si wafer in the reactor established a deposition rate of ∼0.15 nm per cycle with the rate verified by transmission electron microscopy (TEM) measurements on samples of both *nano*ITO and *nano*ITO**|–RuP^2+^** (Fig. S2†). Conformal films were observed on both substrates, suggesting that the adsorbed dye does not hinder conformal Al_2_O_3_ deposition.

**Fig. 2:**
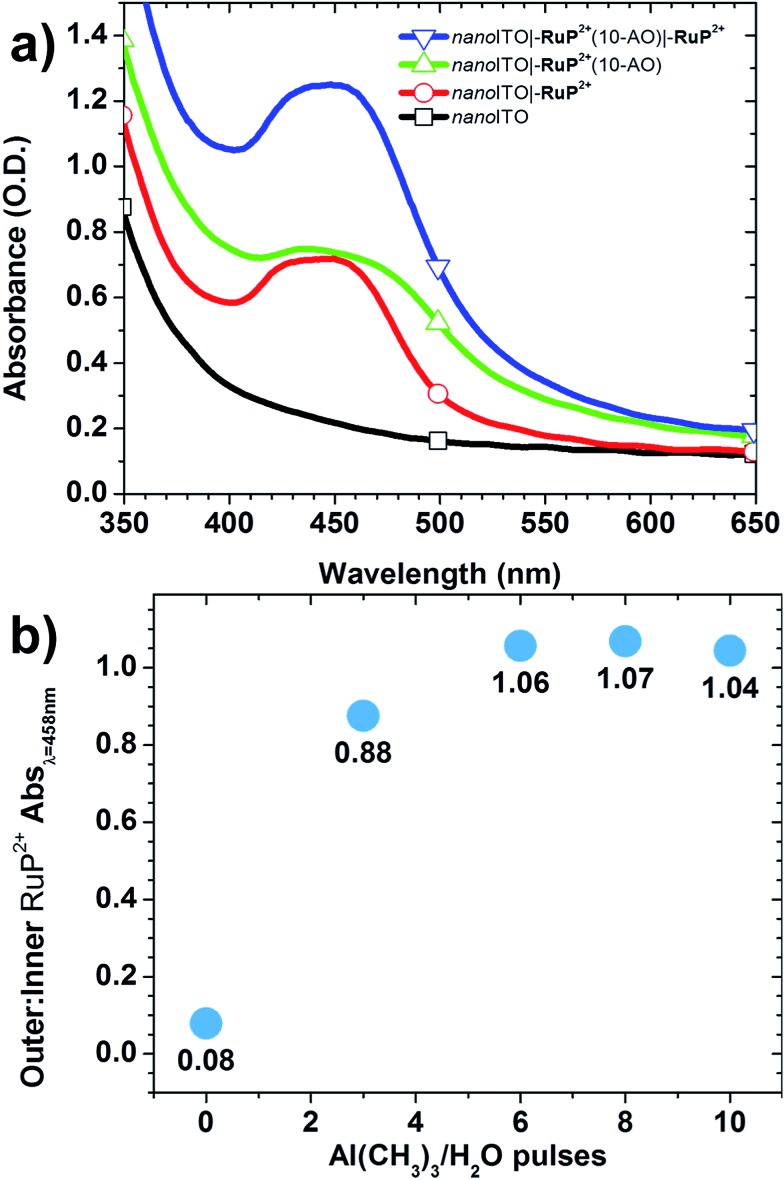
(a) Absorption spectra of dry films from the sequential loading procedure leading to *nano*ITO**|–RuP^2+^**(10-AO)**|–RuP^2+^**; (b) outer : inner **–RuP^2+^** ratios as a function of the number of ALD pulses evaluated at 458 nm with background subtraction.

The effect of additional ALD cycles on sequential loading of a second chromophore layer was investigated by UV-visible absorption measurements. In these experiments, *nano*ITO**|–RuP^2+^** electrodes were subjected to an increasing number of Al(CH_3_)_3_/H_2_O cycles, *x* with *x* = 0, 3, 6, 8, and 10, followed by overnight soaking in the **RuP^2+^** loading solution. UV-visible spectra were used to monitor the surfaces after each step in the surface synthesis (Fig. 2a). The ratio of **RuP^2+^** in the outer layer to **RuP^2+^** in the inner layer was evaluated by taking the ratios of background-subtracted spectra before and after the second loading step. Outer-to-inner ratios at 458 nm are shown in Fig. 2b. The extent of addition of the second **RuP^2+^** layer was dependent on the number of Al(CH_3_)_3_/H_2_O cycles with a ∼1 : 1 ratio reached at 6 cycles and comparable results obtained for 8 and 10 cycles.

Further Al_2_O_3_ addition (20 cycles total) caused a decrease in the 1 : 1 outer-to-inner chromophore loading ratio. UV-visible absorption measurements comparing outer-to-inner loading on a sample of *nano*TiO_2_**|–RuP^2+^**(20-AO)**|–RuP^2+^** showed that loading of the outer chromophore was ∼65% that of the inner chromophore (Fig. S3†). The decrease in loading could be due to reduced pore size and/or a reduced surface area of the films due to Al_2_O_3_ deposition.

To investigate pore size further, BET desorption isotherms were used to determine the pore size distribution of modified and unmodified *nano*ITO films for both *nano*ITO and *nano*ITO(20-AO) (Fig. S4†). The mean pore size decreased by ∼5 nm upon addition of 20-AO, from 36 nm for *nano*ITO to 31 nm for *nano*ITO(20-AO). This decrease is in good agreement with the expected value (6 nm; 20 cycles at 0.15 nm per cycle on each particle), and could explain the decrease in chromophore loading reflecting waning of nanoparticle voids.

The role of ALD overlayer thickness on the photostability of **RuP^2+^** surface-bound to *nano*TiO_2_ and on the electrochemical stability of the water oxidation catalyst, [Ru(2,6-bis(1-methyl-1*H*-benzo[*d*]imidazol-2-yl)pyridine)(4,4′-((HO)_2_(O)P–CH_2_)_2_-2,2′-bipyridine)(OH_2_)]^2+^ (**RuCP(OH_2_)^2+^**, Fig. 1b) on *nano*ITO has been investigated previously.^21^,^22^ For both, maximum stability was achieved for ALD overlayer thicknesses approaching the molecular diameter of **–RuP^2+^** (∼1.3 nm). In synthesizing the chromophore-catalyst assembly, an initial ALD overlayer of 10 Al(CH_3_)_3_/H_2_O cycles (∼1.5 nm) was used to stabilize surface-bound **–RuP^2+^**. In a second step, **RuCP(OH_2_)^2+^** (Fig. 1b), as the trifluoromethanesulfonate salt, was loaded from methanol (∼1 mM in complex) onto the pre-deposited Al_2_O_3_ overlayer coating surface-bound **–RuP^2+^**. In a final step, an additional 10 Al(CH_3_)_3_/H_2_O cycles were deposited to stabilize catalyst surface binding. The second deposition step increased the total thickness of the Al_2_O_3_ overlayer to ∼3 nm, “mummifying” the inner **–RuP^2+^** complex by addition of Al_2_O_3_ to a level that was approximately twice the molecular diameter, note Fig. 1c.

### Electrochemical characterization

**Fig. 3:**
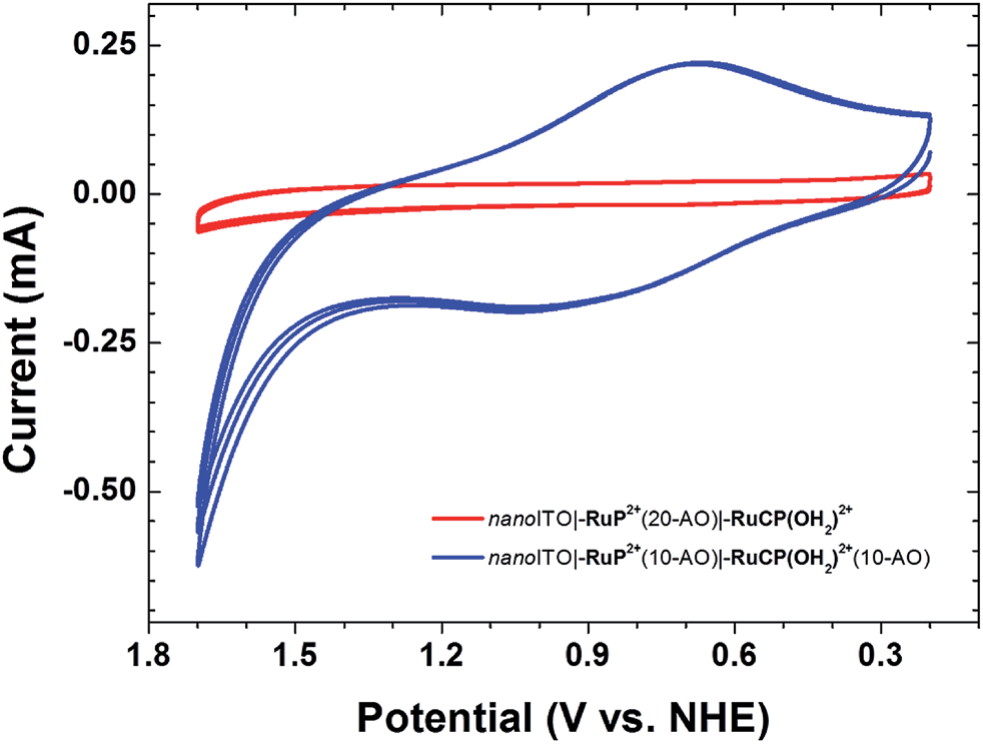
CV scans on *nano*ITO**|–RuP^2+^**(10-AO)**|–RuCP(OH_2_)^2+^**(10-AO) (blue trace) and *nano*ITO**|–RuP^2+^**(20-AO)**|–RuCP(OH_2_)^2+^** (red trace) (conditions: pH 4.7 aqueous sodium acetate (0.1 M); 0.5 M NaClO_4_; *ν* = 20 mV s^–1^; ref = Ag/AgCl; Aux = Pt-mesh).

In cyclic voltammetric (CV) scans on the assembly *nano*ITO**|–RuP^2+^**(10-AO)**|–RuCP(OH_2_)^2+^**(10-AO) at pH 4.7 in an aqueous sodium acetate buffer (*I* = 0.1 M; 0.5 M NaClO_4_) at a scan rate of 20 mV s^–1^, a broad (Δ*E*_p_ ≈ 0.18 V) wave at *E*_1/2_ = 0.73 V *vs.* NHE appears for the external **–Ru^III^CP(OH)^2+^**/**–Ru^II^CP(OH_2_)^2+^** couple (Fig. 3, blue trace); this couple is known to have *E*_1/2_ = 0.75 V at pH 5 on *nano*ITO.^24^ Further oxidation with appearance of the **–Ru^IV^CP(O)^2+^**/**–Ru^III^CP(OH)^2+^** couple at *E*_1/2_ = 1.0 V at pH 5 is not observed on the CV timescale. The inhibition is due to a kinetic effect arising from the proton-coupled electron transfer (PCET) nature of the couple and the insulating Al_2_O_3_ overlayer.^24^

For the assembly, *nano*ITO**|–RuP^2+^**(20-AO)**|–RuCP(OH_2_)^2+^**, with 20 Al_2_O_3_ inner layers, there was no electrochemical response at 20 mV s^–1^ (Fig. 3, red trace). The loss of electrochemical activity is presumably due both to the inability of the “buried” chromophore to achieve charge compensation on the time scale of the experiment upon oxidation to **–RuP^3+^**, 
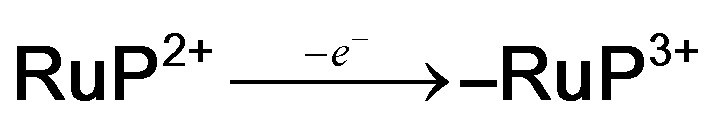
, and to slow electron transfer tunnelling from the external **–RuCP(OH_2_)^2+^** catalyst to the electrode surface.

Oxidation of the external **–RuCP(OH_2_)^2+^** catalyst is influenced by the internal chromophore and continues to occur even with an intervening layer of Al_2_O_3_ without direct surface binding of the chromophore to the underlying *nano*ITO. This effect was demonstrated by CV measurements on an assembly prepared by first depositing 10 layers of Al_2_O_3_ on *nano*ITO followed by surface preparation of the assembly as described above. In CV scans of the resulting assembly, *nano*ITO(10-AO)**|–RuP^2+^**(10-AO)**|–RuCP(OH_2_)^2+^**(10-AO), a broad wave appeared at *E*_1/2_ ≈ 0.64 V at pH 8.8 in a H_2_PO_4_^–^/HPO_4_^2–^ buffer for the **–Ru^III^CP(OH)^2+^**/**–Ru^II^CP(OH_2_)^2+^** couple (Fig. S5†) even though the catalyst couple was separated from the surface by 20 cycles (∼3 nm) of Al_2_O_3_.

A spectroelectrochemical experiment was conducted to resolve the broad, overlapping waves for *nano*ITO(10-AO)**|–RuP^2+^**(10-AO)**|–RuCP(OH_2_)^2+^**(10-AO), the surface-separated mummy sample (Fig. S6†). Slow, 180 s electrochemical steps at 0.02 V increments from 0 to 1.7 V *vs.* NHE with spectrophotometric monitoring revealed a distinct oxidation at *E*_1/2_ = 0.66 V for the **–Ru^III^CP(OH)^2+^**/**–Ru^II^CP(OH_2_)^2+^** couple, in agreement with the CV data. A second oxidation was revealed at *E*_1/2_ = 1.30 V for the **–RuP^3+/2+^** couple which was not observed in CV scans at scan rates as slow as 20 mV s^–1^ because of its kinetic inhibition.^9^ The spectroelectrochemical results confirm that both chromophore and catalyst are redox active with an important role for long-range electron transfer through Al_2_O_3_ mediated by the intervening **–RuP^2+^**.

### Photoelectrochemical hydroquinone dehydrogenation

The “mummy” protected assembly *nano*TiO_2_**|–RuP^2+^**(10-AO)**|–RuCP(OH_2_)^2+^**(10-AO) was investigated as a DSPEC photoanode on *nano*TiO_2_. In these experiments a two-compartment cell with a Nafion membrane separator was used with a three-electrode configuration (SCE reference electrode, Pt-mesh counter electrode). The experiments were conducted under N_2_ at pH 4.7 in a 0.1 M aqueous sodium acetate buffer in 0.5 M NaClO_4_ with a ∼100 mW cm^–2^ white light source (400 nm long-pass filter). An applied bias of 0.24 V *vs.* NHE was used to maximize the photocurrent response.

**Fig. 4:**
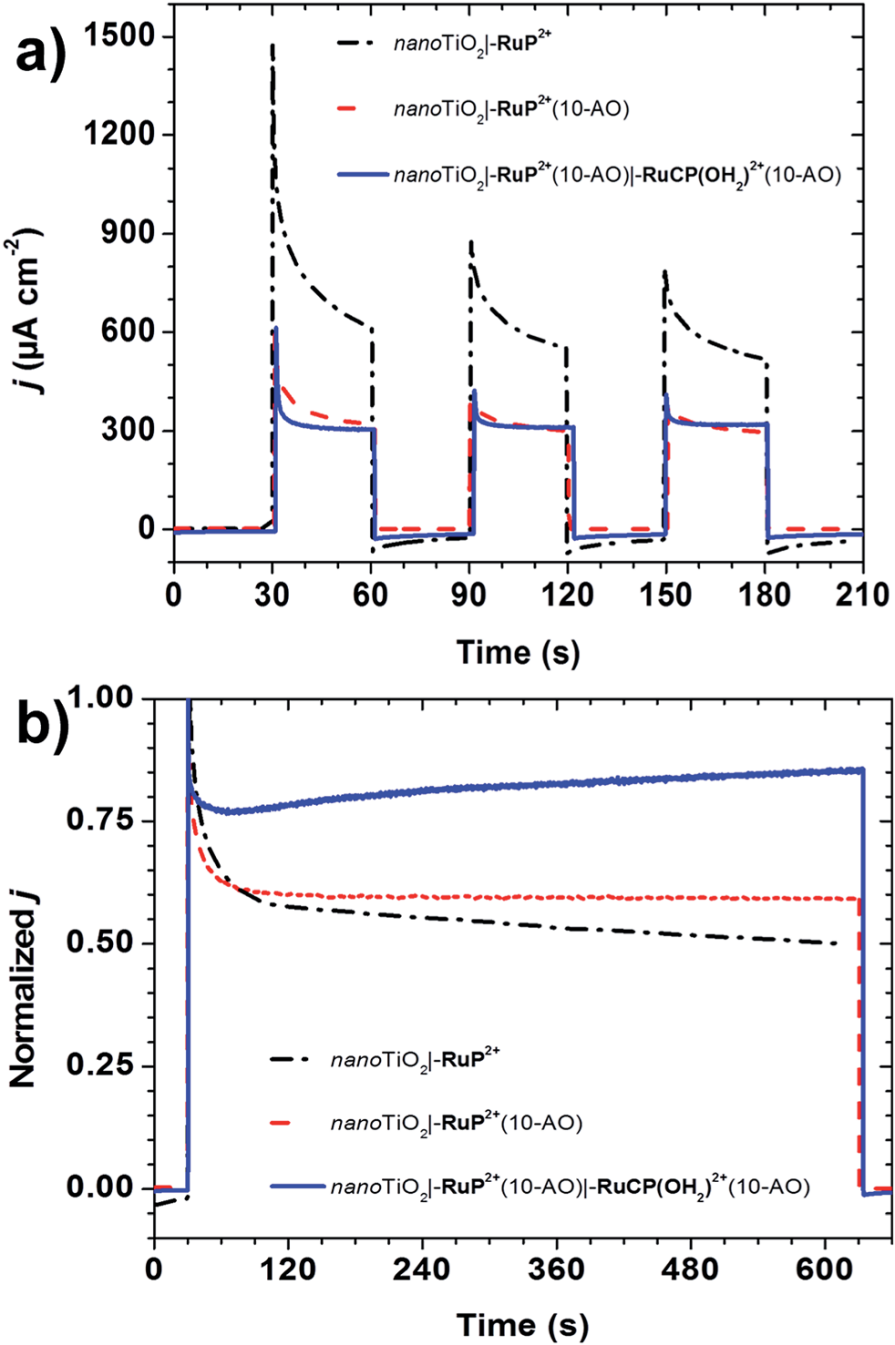
(a) Off–on photocurrent–time traces and; (b) normalized photocurrent–time traces under continuous illumination for 10 minutes for *nano*TiO_2_**|–RuP^2+^** (black dash-dot traces), *nano*TiO_2_**|–RuP^2+^**(10-AO) (red dash traces), and *nano*TiO_2_**|–RuP^2+^**(10-AO)**|–RuCP(OH_2_)^2+^**(10-AO) (blue solid traces) with 20 mM added hydroquinone (conditions: ∼100 mW cm^–2^ white light; *E*_applied_ = 0.24 V *vs.* NHE; pH 4.7 aqueous sodium acetate (0.1 M); 0.5 M NaClO_4_; ref = SCE; Aux = Pt-mesh).

In an initial set of experiments, the photoelectrochemical response of *nano*TiO_2_**|–RuP^2+^**(10-AO)**|–RuCP(OH_2_)^2+^**(10-AO) with added hydroquinone (H_2_Q; 20 mM), added as a sacrificial electron donor (Fig. 4a), was compared to *nano*TiO_2_**|–RuP^2+^**. Under these conditions, excitation and injection by *nano*TiO_2_|–RuP^2+^* is followed by rapid reduction of *nano*TiO_2_(e^–^)**|–RuP^3+^** to *nano*TiO_2_(e^–^)**|–RuP^2+^** by H_2_Q (eqn (1)).

1 TiO_2_**|–Ru(****iii****)P**^**3+**^ + 1/2H_2_Q → TiO_2_**|–Ru(****ii****)P**^**2+**^ + 1/2Q + H^+^

For *nano*TiO_2_**|–RuP^2+^**, a large initial photocurrent spike of ∼1.5 mA cm^–2^ was observed, arising from surface oxidation of the complex and local capacitance effects, with the photocurrent reaching 0.61 mA cm^–2^ after 30 seconds. Under the same conditions, illumination of *nano*TiO_2_**|–RuP^2+^**(10-AO) resulted in an initial current spike of 0.60 mA cm^–2^ decreasing to 0.32 mA cm^–2^ after 30 seconds. The diminished photocurrent is presumably due to the Al_2_O_3_ lowering injection yield as discussed previously.^21^

A nearly identical response was observed for the mummified assembly *nano*TiO_2_**|–RuP^2+^**(10-AO)**|–RuCP(OH_2_)^2+^**(10-AO), with the photocurrent spike reaching 0.61 mA cm^–2^, falling to 0.31 mA cm^–2^ after 30 seconds. This photocurrent response for the mummified assembly, in which the chromophore is fully buried by Al_2_O_3_, points to injection by –RuP^2+^* and hole transfer from **–RuP^3+^** to the catalyst in the outer-layer followed by reduction of **–Ru^III^CP(OH)^2+^** by H_2_Q (eqn (2) and (3)). To validate this explanation, photocurrents for *nano*TiO_2_**|–RuP^2+^**(20-AO), *nano*TiO_2_**|–RuP^2+^**(20-AO)**|–RuCP(OH_2_)^2+^**), and *nano*TiO_2_**|–RuCP(OH_2_)^2+^**(10-AO) were all negligible at <0.02 mA (Fig. S7†). These results point to the importance of the chromophore and the configuration of the mummy-protection in obtaining a significant level of photoelectrochemical activity.

2 TiO_2_**|–Ru(****iii****)P**^**3+**^**|–Ru(****ii****)CP(OH**_**2**_**)**^**2+**^ → TiO_2_**|–Ru(****ii****)P**^**2+**^**|–Ru(****iii****)CP(OH)**^**2+**^ + H^+^

3 TiO_2_**|–Ru(****ii****)P**^**2+**^**|–Ru(****iii****)CP(OH)**^**2+**^ + 1/2H_2_Q → TiO_2_**|–Ru(****ii****)P**^**2+**^**|–Ru(****ii****)CP(OH**_**2**_**)**^**2+**^ + 1/2Q

Longer-term photolyses were undertaken to assess the impact of ALD stabilization on photocurrent performance. Photocurrent–time traces, normalized to their respective initial current spikes, are shown in Fig. 4b. The photocurrent response for *nano*TiO_2_**|–RuP^2+^** decreased to ∼57% of the maximum value after two minutes of photolysis with a further decrease to ∼50% after ten minutes. With ALD stabilization in *nano*TiO_2_**|–RuP^2+^**(10-AO), the photocurrent decreased to ∼60% after two minutes but with less than a 1% decrease between two and ten minutes. This comparison highlights the importance of the ALD overlayer in *nano*TiO_2_**|–RuP^2+^**(10-AO) in inhibiting loss of **–RuP^2+^** from the surface and, with the addition of H_2_Q, rapid reduction of **–RuP^3+^** to **–RuP^2+^** in avoiding its decomposition on the surface.^18^

By contrast, for the mummy-protected assembly, *nano*TiO_2_**|–RuP^2+^**(10-AO)**|–RuCP(OH_2_)^2+^**(10-AO), the normalized photocurrent response *increased* from 79% to 85% over the final eight minutes of illumination. This “breaking in” period arises from hydrolysis of an alumina adduct with the catalyst which forms during the ALD process. The adduct forms following exposure of oxide-bound **–RuCP(OH_2_)^2+^** to pulses of Al(CH_3_)_3_ without subsequent re-coordination of the aquo as evidenced by a ∼1600 cm^–1^ red shift in the visible MLCT *λ*_max_ from 487 to 530 nm and a noticeable color change on the surface (Fig. S8†). Subsequent oxidative CV scans through the Ru^III/II^ wave (Fig. S9†), or photoelectrolysis cycles, at pH 4.7 in an aqueous sodium acetate buffer (*I* = 0.1 M; 0.5 M NaClO_4_), restore the aquo form of the catalyst, **–Ru^III^CP(OH)^2+^**. The photocurrent enhancement is due to an enhanced rate of H_2_Q oxidation by the oxidized catalyst compared to **–RuP^2+^**.^25^

### Electrocatalytic water oxidation

Electrocatalytic water oxidation was investigated for *nano*ITO**|–RuP^2+^**(10-AO)**|–RuCP(OH_2_)^2+^**(10-AO) with *nano*ITO**|–RuP^2+^**(10-AO) as a control with the same cell configuration as in the photoelectrochemical experiments. Electrolyses were carried out at pH 8.8 sodium phosphate dibasic (*I* = 0.1 M; 0.4 M NaClO_4_). O_2_ was detected by using a parallel collector–generator electrode technique (see Experimental section) with real-time detection of O_2_ at –0.61 V *vs.* NHE.^11^,^26^,^27^ The potential at the working electrode was first held at 0 V *vs.* NHE for two hours to simulate the dark-current background and reduce trace O_2_ in the cell.

Water oxidation was initiated by stepping the electrode potential to *E*_app_ = 1.4 V *vs.* NHE, past *E*_1/2_ = 1.3 V for the **–RuP^3+/2+^** couple with the electrolysis continued for two hours.

**Fig. 5:**
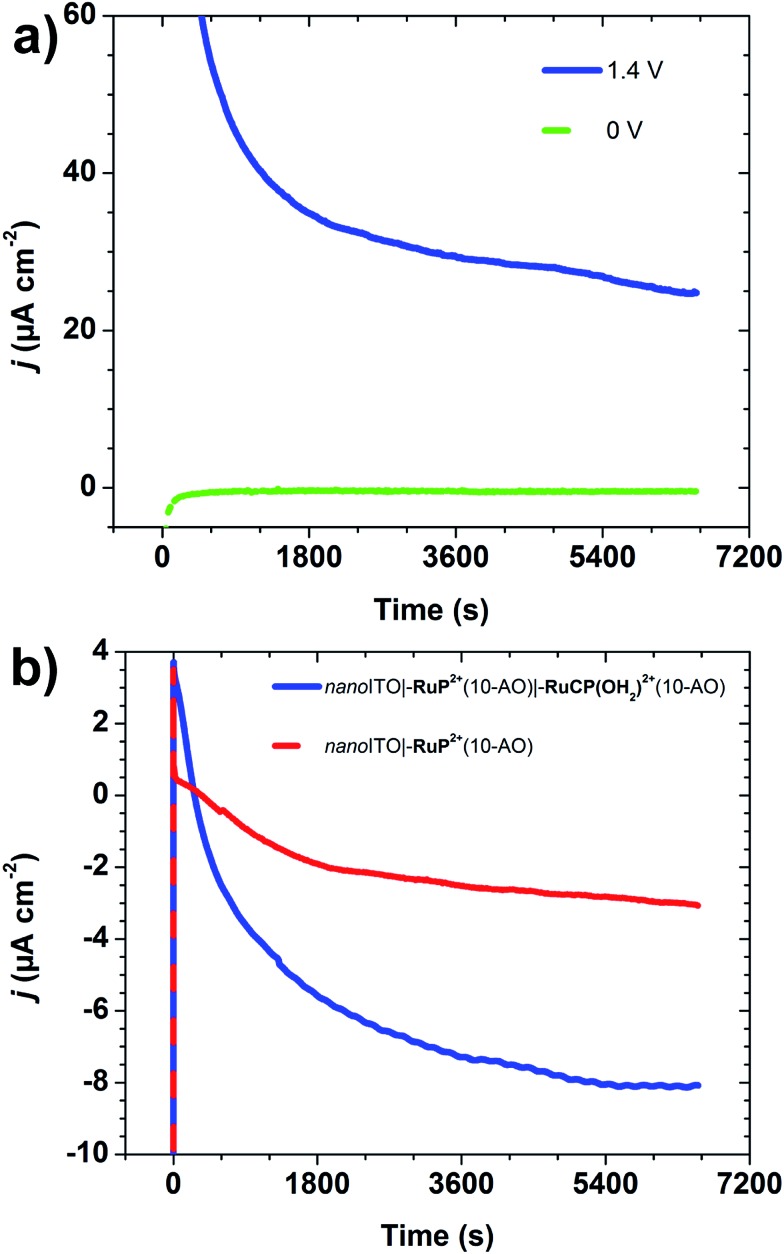
(a) Current–time traces for *nano*ITO**|–RuP^2+^**(10-AO)**|–RuCP(OH_2_)^2+^**(10-AO) with *E*_gen_ = 1.4 V (blue trace) and 0 V *vs.* NHE (green trace); (b) background (*i.e. E*_gen_ = 0 V *vs.* NHE)-subtracted current–time traces for the FTO collector electrode for *nano*ITO**|–RuP^2+^**(10-AO)**|–RuCP(OH_2_)^2+^**(10-AO) (blue trace) and *nano*ITO**|–RuP^2+^**(10-AO) (red trace), with *E*_coll_ = –0.61 V *vs.* NHE. Cathodic currents arise from O_2_ reduction at the FTO collector electrode (conditions: pH 8.8, 0.1 M H_2_PO_4_^–^/HPO_4_^2–^; 0.4 M NaClO_4_; ref = SCE; Aux = Pt-mesh).

The appearance of a significant catalytic current at *E*_app_ = 1.4 V in the current–time trace in Fig. 5 is notable, because the onset potential for water oxidation catalysis by **–RuCP(OH_2_)^2+^** is known to occur at ∼1.6 V, near *E*_1/2_ for the –Ru^V^(O)^3+/2+^ catalyst couple.^27^ As found earlier for a surface-bound chromophore-catalyst assembly, the low potential onset may be due to concerted electron-atom proton transfer with O-atom transfer to a water molecule accompanied by single electron transfer to both **–RuP^3+^** and **–Ru^IV^CP(O)^2+^** and proton transfer to an external base.^6^,^22^

Currents of >60 μA cm^–2^ were obtained at *E*_app_ = 1.4 V which slowly decreased to >20 μA cm^–2^ over a 2 h period. Water oxidation catalysis was verified by O_2_ detection at the collector electrode compared to the control sample (Fig. 5b). Integration of current passed resulted in a Faradaic efficiency for O_2_ evolution of ∼23% with the origin of loss presumably due to competitive decomposition of the polypyridyl ligand in the **–Ru^IV^CP(O)^2+^** form of the catalyst as reported earlier for a related complex.^28^

As calculated by eqn (4), the turnover frequency (TOF) for water oxidation was 0.014 s^–1^ at *E*_app_ = 1.4 V. In eqn (4), *Q*_O_2_ reduction_ (C) is the integrated charge passed for O_2_ reduction at the FTO collector electrode, *Г* (mol cm^–2^) is the surface coverage of **RuCP(OH_2_)^2+^**, *F* is Faraday's constant (96 485 C mol^–1^), *n*_cat_ = 4 is the electrochemical stoichiometry for water oxidation to O_2_, *η*_collection_ = 0.7 is the collection efficiency at the collector electrode,^11^*A* (cm^2^) is the exposed area of the electrode, and *t* (s) is the electrolysis time. This estimate is a lower limit for water oxidation since surface coverages (*Γ*) were evaluated by UV-visible measurements and not all of the catalytic sites may be electrochemically active due to the Al_2_O_3_ overlayer. For comparison, for a closely related chromophore-catalyst assembly with the same catalyst but prepared by an electro-assembly technique, the TOF was 0.046 s^–1^ at *E*_app_ = 1.7 V in a pH 4.7 aqueous sodium acetate buffer (*I* = 0.1 M; 0.5 M NaClO_4_), but with **–Ru^V^CP(O)^3+^** as the active oxidant rather than **–Ru^IV^CP(O)^2+^**.^10^

4 TOF = *Q*_O_2_ reduction_/(*n*_cat_*FAΓtη*_collection_)

### Photoelectrochemical water oxidation

Photoelectrochemical water oxidation was investigated for *nano*TiO_2_**|–RuP^2+^**(10-AO)**|–RuCP(OH_2_)^2+^**(10-AO) by using the same cell configuration as in hydroquinone dehydrogenation studies. The experiments were conducted in pH 8.8 sodium dibasic phosphate (*I* = 0.1 M; 0.4 M NaClO_4_) with O_2_ detection by the parallel collector–generator technique described earlier.

**Fig. 6:**
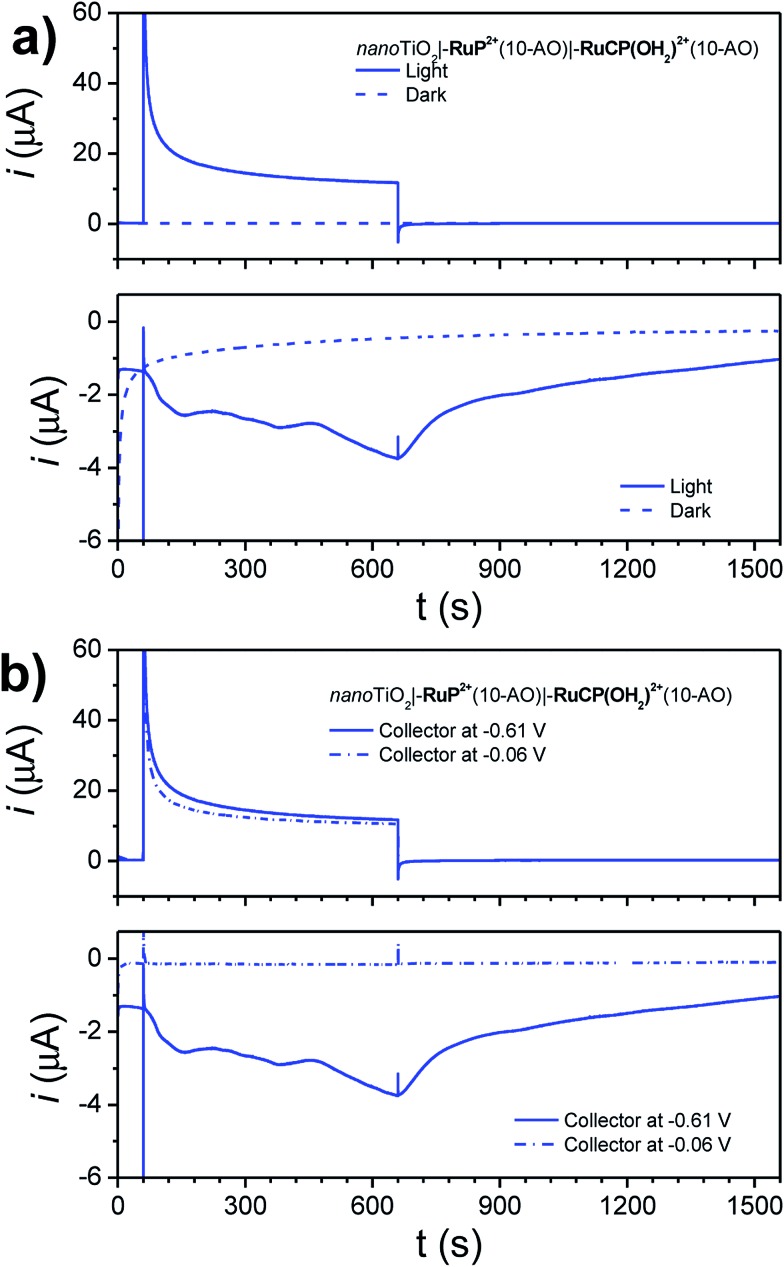
(a) Photocurrent–time traces for *nano*TiO_2_**|–RuP^2+^**(10-AO)**|–RuCP(OH_2_)^2+^**(10-AO) at the (top) generator electrode and (bottom) collector electrode under illumination (solid traces) and in the dark (dashed traces) with *E*_gen_ = 0.64 V *vs.* NHE and *E*_coll_ = –0.61 V *vs.* NHE. (b) Photocurrent–time traces for *nano*TiO_2_**|–RuP^2+^**(10-AO)**|–RuCP(OH_2_)^2+^**(10-AO) at the (top) generator electrode and (bottom) collector electrode under illumination with *E*_gen_ = 0.64 V *vs.* NHE and *E*_coll_ = –0.61 V *vs.* NHE (solid traces) or *E*_coll_ = –0.06 V *vs.* NHE (dashed traces) (conditions: ∼200 mW cm^–2^ white light illumination; 380 nm long-pass filter; pH 8.8, 0.1 M H_2_PO_4_^–^/HPO_4_^2–^; 0.4 M NaClO_4_; ref = SCE; Aux = Pt-mesh).

Short illumination periods (15 minutes) with an intense white light source (∼200 mW cm^–2^, 380 nm long-pass filter) resulted in the photocurrent responses shown in Fig. 6 (*E*_gen_ = 0.64 V; *E*_coll_ = –0.61 V *vs.* NHE).

Upon illumination, instantaneous photocurrent is produced at the photoanode (generator) electrode. A cathodic current is gradually observed at the collector electrode, indicative of O_2_ reduction following diffusion from the generator. Two control assemblies (a non-ALD-protected chromophore-catalyst and a chromophore-only photoanode) did not show productive O_2_ current at the collector electrode (Fig. S10†)).

To ensure the cathodic current at the collector electrode was not due to desorbing, oxidized Ru species, the potential at the collector electrode was raised from –0.61 V to –0.06 V. A potential of –0.06 V is sufficiently negative to reduce Ru(iii) → Ru(ii), but not sufficient for O_2_ reduction. As seen in Fig. 6b, despite similar a similar photocurrent response at the photoanode generator, no corresponding cathodic current was observed at the collector electrode, suggesting that the cathodic current observed previously is not due to diffusing Ru(iii) species.

**Fig. 7:**
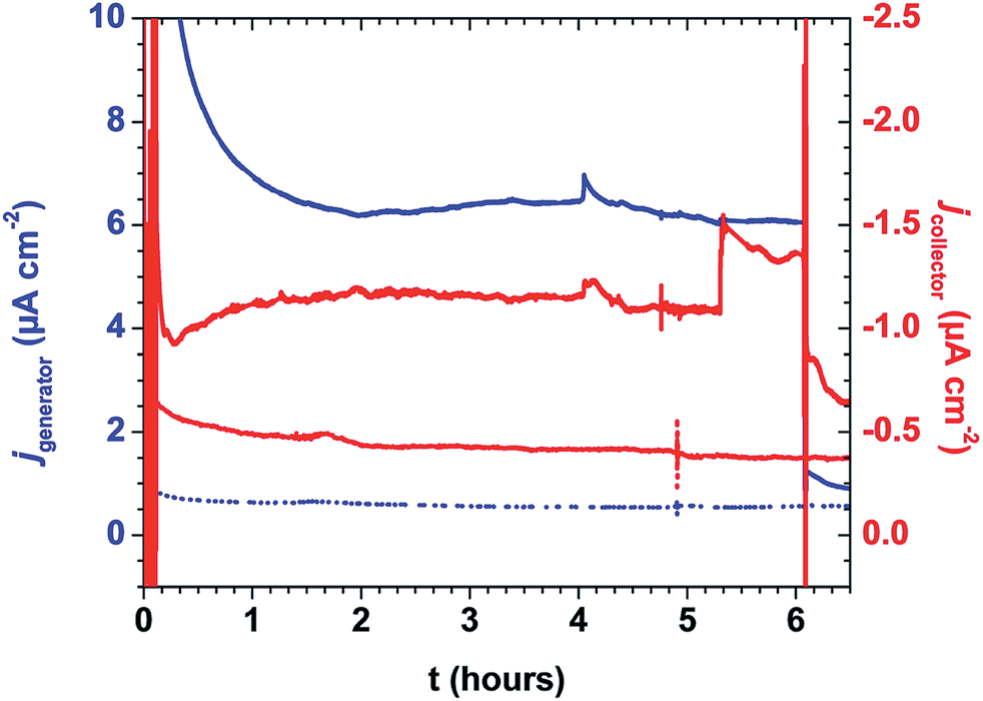
Current–time traces for *nano*TiO_2_**|–RuP^2+^**(10-AO)**|–RuCP(OH_2_)^2+^**(10-AO) *E*_gen_ = 0.64 V and *E*_coll_ = –0.61 V under illumination. Blue traces (left axis) indicate generator current under illumination (solid) and in the dark (dotted) while red traces (right axis) indicate collector current with the same convention. Cathodic current arises from O_2_ reduction at the FTO collector electrode (conditions: ∼200 mW cm^–2^ white light illumination; 400 nm long-pass filter; pH 8.8, 0.1 M H_2_PO_4_^–^/HPO_4_^2–^; 0.4 M NaClO_4_; ref = SCE; Aux = Pt-mesh).

An extended photoelectrolysis (6 hours) was performed on the mummified assembly (Fig. 7). Under intense white light (∼200 mW cm^–2^, 400 nm long-pass filter), the assembly showed sustained generator and collector current over the course of the 6 hour illumination. The generator current decayed instantaneously upon shuttering the light, while the collector current gradually decayed, similar to the current traces observed over shorter time periods. Integration of the current passed allowed for a comparison of the cumulative Faradaic efficiency as a function of time (Fig. S11†) by the following equation:
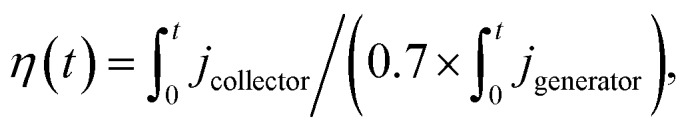
where 0.7 is the collection efficiency at the collector electrode, and *t* is the time (s) of illumination. Over the course of illumination, the Faradaic efficiency is observed to increase, ultimately reaching 16.8% after 6 hours. As a comparison, a recently reported electro-assembled chromophore-catalyst assembly showed a Faradaic efficiency of 8% for O_2_ production for light-assisted water oxidation (100 mW cm^–2^, 380 nm cut-off filter after 10 minutes of illumination; 4.8% after 10 minutes in the mummified system).^11^ This comparison suggests that mummified/ALD-constructed assemblies compare favourably to chromophore-catalyst assemblies constructed by other reported methods.

## Conclusions

We describe here a novel procedure for the direct surface preparation of chromophore-catalyst assemblies based on phosphonate surface binding and ALD deposition of Al_2_O_3_ overlayers. It features high surface stability and electronically linked chromophore and catalyst pairs without covalent bond formation with an ALD mummy strategy for stabilizing the surface-bound chromophore. Although electron transfer is inhibited on the ALD stabilized surfaces, they do undergo injection and assembly oxidation with sustained photocurrents observed in a DSPEC with added hydroquinone. Electrocatalytic water oxidation is also observed for the mummy assembly with sustained catalytic currents at applied potentials below those required for oxidation of the catalyst to **–Ru^V^CP(O)^3+^**, apparently by intervention of a concerted electron-atom transfer pathway observed earlier in a covalently linked assembly. Light-assisted water oxidation catalysis has been observed over a continuous 6 hour illumination period. Experiments incorporating a more active catalyst are currently underway.

## Experimental

### Materials and methods

#### Materials

De-ionized water was further purified using a Milli-Q Ultrapure water purification system. Additional solvents, hydrochloric acid, and glacial acetic acid were purchased from Fisher Scientific and were used as received. Sodium acetate, sodium phosphate (monobasic, anhydrous), and sodium phosphate dibasic (anhydrous) were purchased from Sigma-Aldrich, were ACS Reagent grade or better, and were used as received. [Ru(Mebimpy)(Cl)(μ-Cl)]_2_ (Mebimpy = 2,6-bis(1-methyl-1*H*-benzo[*d*]imidazol-2-yl)pyridine),^10^ 4,4′-((HO)_2_(O)P–CH_2_)_2_-2,2′-bipyridine,^23^*cis*-[Ru(2,2′-bipyridine)_2_(Cl)_2_],^9^ and 4,4′-((EtO)_2_(O)P)_2_-2,2′-bipyridine^23^ were synthesized according to literature protocols. Fluorine-doped tin oxide (FTO, 15 Ω per square sheet resistance) was purchased from Hartford Glass (Hartford City, IN) and was cleaned by sonication in ethanol (20 min), 0.1 M HCl in ethanol (20 min), and ethanol (20 min) prior to use.

### Synthesis of molecular complexes

Synthesis and characterization of **RuP^2+^** and **RuCP(OH_2_)^2+^** as their chloride and trifluoromethanesulfonate salts, respectively, were described previously.^23^,^29^–^31^ Further details are available in the ESI.†

### Nanoparticle TiO_2_ films (*nano*TiO_2_)

Nanoparticles of TiO_2_ were prepared as described previously.^32^,^33^ Nanoparticle paste was spread on FTO glass using the doctor-blade method with 1 layer of Scotch tape (Fig. S12†). Film thicknesses were approximately 4 μm thick.

### Nanoparticle ITO films (*nano*ITO)

Nanoparticles of tin-doped indium oxide (ITO, TC8 DE; 20 wt% dispersion in ethanol) were purchased from Evonik Industries and were prepared as described previously.^34^ Nanoparticle paste was spread on FTO glass using the doctor-blade method with 1 layer of Scotch tape (Fig. S12†). Film thicknesses were approximately 4 μm thick.

### BET measurements

The mean pore sizes of *nano*ITO and *nano*ITO(20-AO) were determined using BET desorption isotherms. The electrodes were cut into small (∼0.07 cm^2^) pieces and placed in a BET sample bulb. The *nano*ITO nanoparticles were not removed from the FTO glass substrate so as to preserve the pore structure, while all glass scoring was made to the backside of the FTO glass substrate. Approximately 8 g of material (which represents a projected area of ∼8 cm^2^) was placed in the sample bulb and was heated to 140 °C under vacuum for 22 h using a Quantachrome NOVA 200 system. The samples and sample bulbs cooled to room temperature, after which they were back-filled with helium. Given the majority of the sample mass was FTO glass, specific surface area measurements were not revealing. Using the desorption isotherms with 0.6 < *P*/*P*_o_ < 0.95, the pore size distribution was determined by Barrett–Joyner–Halenda (BJH) analysis. The mean pore sizes for *nano*ITO and *nano*ITO(20-AO) were 36 nm and 31 nm, respectively.

### Atomic layer deposition (ALD)

ALD was performed by using a Cambridge NanoTech Savannah S200 ALD system located in the Chapel Hill Analytical and Nanofabrication Laboratory (CHANL) cleanroom. The reactor was set at 150 °C. Prior to deposition, samples sat in the reactor under continuous nitrogen purge (99.999%, further purified using an Entegris GateKeeper Inert Gas Purifier) at 150 °C for a minimum of 10 minutes. Each deposition cycle consisted of a 0.02 s pulse of trimethylaluminum (Al(CH_3_)_3_, 97% purity), a 20 s exposure in the reactor, a 60 s purge, a 0.02 s pulse of water, a 20 s exposure in the reactor, and a 60 s purge.

### FTO collector–generator electrodes

Dual working electrodes were constructed by adapting a technique developed by Mallouk.^11^,^26^ Thin strips of non-conductive glass (∼2–3 mm wide, 1 mm thick) were applied to working electrodes (*nano*TiO_2_ or *nano*ITO) using epoxy (Loctite Hysol E-00CL) and allowed to cure. FTO was then attached using epoxy such that the conductive side of each electrode faced inward (Fig. S1†). The electrolytic solution is drawn between the working electrodes by capillary action.

### Electrochemistry

Cyclic voltammetry (CV) and current–time measurements were performed with a CH Instruments potentiostat (model 601D or 660D) or bipotentiostat (model 760E). Typically, a two-compartment glass cell (working electrode and reference/counter electrodes separated by a fine-porosity glass frit) was used. The reference electrode was Ag/AgCl (3 M NaCl, *E* = 0.2 V *vs.* NHE). The counter electrode was Pt metal (wire or mesh).

### Spectroelectrochemistry

Spectroelectrochemical measurements were performed in a one-compartment glass cuvette with a Ag/AgCl (3 M NaCl) reference electrode and a Pt metal mesh counter electrode. The reference and counter electrodes were placed behind the working electrode such that contact was made with the non-conductive glass. The working electrode (*nano*ITO) was placed at a 45° angle to the path of the beam. UV-visible absorption spectra were collected with an Agilent 8453 UV-visible photodiode array spectrophotometer. The potential of the working electrode was stepped from –0.2 V to 1.5 V *vs.* Ag/AgCl (3 M NaCl) with a potential step every 0.02 V. The potential at each step was held for 180 s to achieve equilibrium. Redox potentials were obtained by fitting using SPECFIT/32 software.

### Electrocatalysis

Electrocatalytic water oxidation experiments were conducted in a two-compartment cell with the working electrode (*nano*ITO-FTO dual electrode, see above) and reference (SCE, *E* = 0.24 V *vs.* NHE)/counter (Pt mesh) electrodes separated by a Nafion membrane. A bipotentiostat (CHI 760E) was used to poise the potential of the working generator (*nano*ITO) electrode at a set potential while the working collector (FTO) electrode was poised at –0.61 V *vs.* NHE for *in situ* reduction of O_2_ as it formed. Prior to electrocatalysis, the buffer solution (pH 8.8, 0.1 M H_2_PO_4_^–^/HPO_4_^2–^ in 0.4 M NaClO_4_) was de-aerated with N_2_ for ∼15 min. A positive stream of N_2_ was maintained in the headspace to avoid atmospheric O_2_ diffusion into the solution. The potential of the working generator electrode was poised at 0 V *vs.* NHE for two hours to approximate a dark current trace before immediately stepping the potential to 1.4 V *vs.* NHE for two hours. Currents were normalized for the geometric areas of the working electrodes.

### Photoelectrochemistry (hydroquinone)

Photoelectrochemical experiments were conducted in a two-compartment cell with the working electrode (*nano*TiO_2_, working area defined by Hysol E-00CL epoxy) and reference (SCE, *E* = 0.24 V *vs.* NHE)/counter (Pt mesh) electrodes separated by a Nafion membrane. A bipotentiostat (CHI 760E) was used to poise the potential of the working electrode (0.24 V *vs.* NHE) to maximize hydrogen evolution at the counter electrode. Prior to illumination, the buffer solution (pH 4.7 HOAc/NaOAc, 0.1 M; 0.5 M NaClO_4_ supporting electrolyte) with added hydroquinone (0.02 M) was de-aerated with N_2_ for ∼15 min. A positive stream of N_2_ was maintained in the headspace to avoid atmospheric O_2_ diffusion into the solution. Illumination was provided by a Thor Labs HPLS-30-04 light source. Samples were positioned to receive ∼100 mW cm^–2^ (1 sun, 400 to 700 nm) with the light intensity determined with an Oriel Instruments 91150V reference cell. A 400 nm long-pass filter was used to prevent direct bandgap excitation of *nano*TiO_2_. Preliminary experiments were simple “off–on” illumination cycles with 30 s intervals of dark followed by illumination. Dark and light *J*–*V* curves were also obtained at a scan rate of 5 mV s^–1^. Continuous illumination was performed for 10 minutes. The photocurrent data were normalized to the initial current spike and for the area of *nano*TiO_2_ illuminated.

### Photoelectrochemistry (water oxidation)

Photoelectrochemical experiments for water oxidation were conducted in a two-compartment cell with the working electrode (*nano*TiO_2_, working area defined by Hysol 608 epoxy) and reference (SCE, *E* = 0.24 V *vs.* NHE)/counter (Pt mesh) electrodes separated by a Nafion membrane. A bipotentiostat (CHI 760E) was used to poise the potential of the working electrode (0.64 V *vs.* NHE, 1.16 V *vs.* RHE) to maximize hydrogen evolution at the counter electrode. Prior to illumination, the buffer solution (pH 8.8 NaH_2_PO_4_/Na_2_HPO_4_, 0.1 M; 0.4 M NaClO_4_ supporting electrolyte) was de-aerated with N_2_ for ∼15 min. A positive stream of N_2_ was maintained in the headspace to avoid atmospheric O_2_ diffusion into the solution. Illumination was provided by a Thor Labs HPLS-30-04 light source. Samples were positioned to receive ∼200 mW cm^–2^ (2 sun, 400 to 700 nm) with the light intensity determined with an Oriel Instruments 91150V reference cell. For short illumination periods (15 minutes), a 380 nm long-pass filter was used to maximize the photoelectrochemical activity. For long illumination periods (2–6 hours), a 400 nm long-pass filter was used to prevent direct bandgap excitation of *nano*TiO_2_.

## Supplementary Material

Supplementary information
