# Supplementary material for: Synthesis, characterization, and water oxidation by a molecular chromophore-catalyst assembly prepared by atomic layer deposition. The “mummy” strategy
Source: Chem Sci. 2015 Jul 31;6(11):6398–406. doi: 10.1039/c5sc01752a (PMC6054119; doi:10.1039/c5sc01752a)
Supplement: Supplementary file 1 [file SC-006-C5SC01752A-s001.pdf]

Electronic Supplementary Material (ESI) for Chemical Science.
This journal is © The Royal Society of Chemistry 2015

Electronic Supporting Information

Title: Synthesis, characterization, and water oxidation by a molecular chromophore-catalyst assembly prepared by atomic layer deposition. The "mummy" strategy.

Authors: Alexander M. Lapides, Benjamin D. Sherman, M. Kyle Brennaman, Christopher J. Dares, Kasey R. Skinner, Joseph L. Templeton, and Thomas J. Meyer

Table of Contents

Materials and Methods....2

Figure S1 – Collector-generator configuration....3

Figure S2 – TEM Images of core/shell ITO/Al_2O_2....4

Figure S3 – Absorption spectra of *nano*TiO_2|-**RuP**$^{2+}$(20-AO)|-**RuP**$^{2+}$, *nano*TiO_2|-**RuP**$^{2+}$, and *nano*TiO_2|(20-AO)|-**RuP**$^{2+}$....5

Figure S4 – Pore size distributions of *nano*ITO and *nano*ITO(20-AO)....6

Figure S5 – CV scan of *nano*ITO(10-AO)|-**RuP**$^{2+}$(10-AO)|-**RuCP(OH$_2$)**$^{2+}$(10-AO)....6

Figure S6 – Spectroelectrochemistry of *nano*ITO(10-AO)|-**RuP**$^{2+}$(10-AO)|-**RuCP(OH$_2$)**$^{2+}$(10-AO)....7

Figure S7 – Photocurrent traces of inactive samples....8

Figure S8 – UV-visible absorption spectra of Al_2O_3-catalyst adduct....8

Figure S9 – CV scans of Al_2O_3-catalyst adduct....9

Figure S10 – Collector-Generator Current-Time traces for *nano*TiO_2|-**RuP**$^{2+}$(10-AO)|-**RuCP(OH$_2$)**$^{2+}$ and *nano*TiO_2|-**RuP**$^{2+}$(10-AO)....9

Figure S11 – Faradaic Efficiency-Time plot for Light-Assisted Water Oxidation of *nano*TiO_2|-**RuP**$^{2+}$(10-AO)|-**RuCP(OH$_2$)**$^{2+}$(10-AO)....10

Figure S12 – SEM Images of *nano*TiO_2 and *nano*ITO films....11

Additional References....11

Materials and Methods:

*Synthesis of **RuP**$^{2+}$.*[1] In a 100-mL Teflon microwave vessel, *cis*-[Ru(2,2'-bipyridine)$_2$(Cl)$_2$] (227 mg, 4.69×10^{-4} mol) and 4,4'-((EtO)$_2$(O)P)$_2$-2,2'-bipyridine (201 mg, 4.69×10^{-4} mol) were suspended in water (17 mL). The vessel was placed in a microwave reactor where, following a 5-minute ramping period, it was heated at 140 °C for 10 minutes. The pressure of the vessel did not exceed 300 PSI. The vessel was allowed to cool to room temperature. The suspension was filtered through a Millex GP PES Membrane (0.22 μm). The solvent was removed from the filtrate on a rotary evaporator. The residue was stirred in refluxing 4 M HCl overnight. The solvent was removed on a rotary evaporator. The resulting residue was triturated with ether and collected over a glass frit. The orange-red precipitate was washed with ether and collected (339 mg, 4.23×10^{-4} mol, 90%).

The ^{1}H NMR spectrum matched that of a previously reported sample.

Analytical HPLC (75:25 water:methanol) of the sample indicated ≥ 95% purity, with the likely impurity being [Ru(2,2'-bipyridine)$_3$][Cl]$_2$, which should not bind to metal oxide electrode surfaces.

*Synthesis of **RuCP(OH$_2$)**$^{2+}$.*[2-4] In a 100-mL Teflon microwave vessel, [Ru(2,6-bis(1-methyl-1*H*-benzo[*d*]imidazol-2-yl)pyridine)(Cl)]$_2$[Cl]$_2$ (41.2 mg, 4.03×10^{-5} mol) and 4,4'-(H$_2$O$_3$P-CH$_2$)$_2$-2,2'-bipyridine (27.8 mg, 8.08×10^{-5} mol) were suspended in ethanol (20 mL) and water (10 mL). The vessel was briefly subject to sonication (~1 minute). The vessel was placed in a microwave reactor where, following a 5-minute ramping period, it was heated at 160 °C for 30 minutes. The pressure of the vessel did not exceed 300 PSI. The vessel was allowed to cool to room temperature. The solution was filtered. From the filtrate, the solvent was removed on a rotary evaporator. The residue was dried under vacuum overnight. To the residue, anhydrous methylene chloride (50 mL) was added. The suspension was de-aerated with argon for 15 minutes. With a vent needle in place and under continuous flow of argon (CAUTION: HCl gas is evolved), trifluoromethanesulfonic acid (1.8 mL) was added slowly. The reaction immediately releases HCl (gas). The reaction stirred at room temperature overnight (note: the flow of argon was high enough to vent HCl but low enough as to not evaporate methylene chloride over the time of the experiment). Following the reaction, diethyl ether was added to precipitate the solid, which was collected on a glass frit and washed with diethyl ether (62.4 mg, 5.67×10^{-5} mol, 70%).

The ^{1}H NMR spectrum matched that of a previously reported sample.

Analytical HPLC (40:60 water:methanol) of the sample revealed three product peaks; however the UV-visible absorption spectrum of each peak was identical.

Complex Loading. Methanol solutions (~1 mM) of **RuP**$^{2+}$ and **RuCP(OH$_2$)**$^{2+}$ were used for complex loading. Electrodes (*nano*ITO or *nano*TiO$_2$) were placed in the solutions to load complexes, typically overnight. Following loading, the electrodes were rinsed with methanol and dried under a stream of air or N$_2$. Surface coverage was determined using the background-subtracted absorbance values from UV-visible absorption spectra and the following equation: $\Gamma = A(\lambda)\times(\epsilon(\lambda)\times1000)^{-1}$.

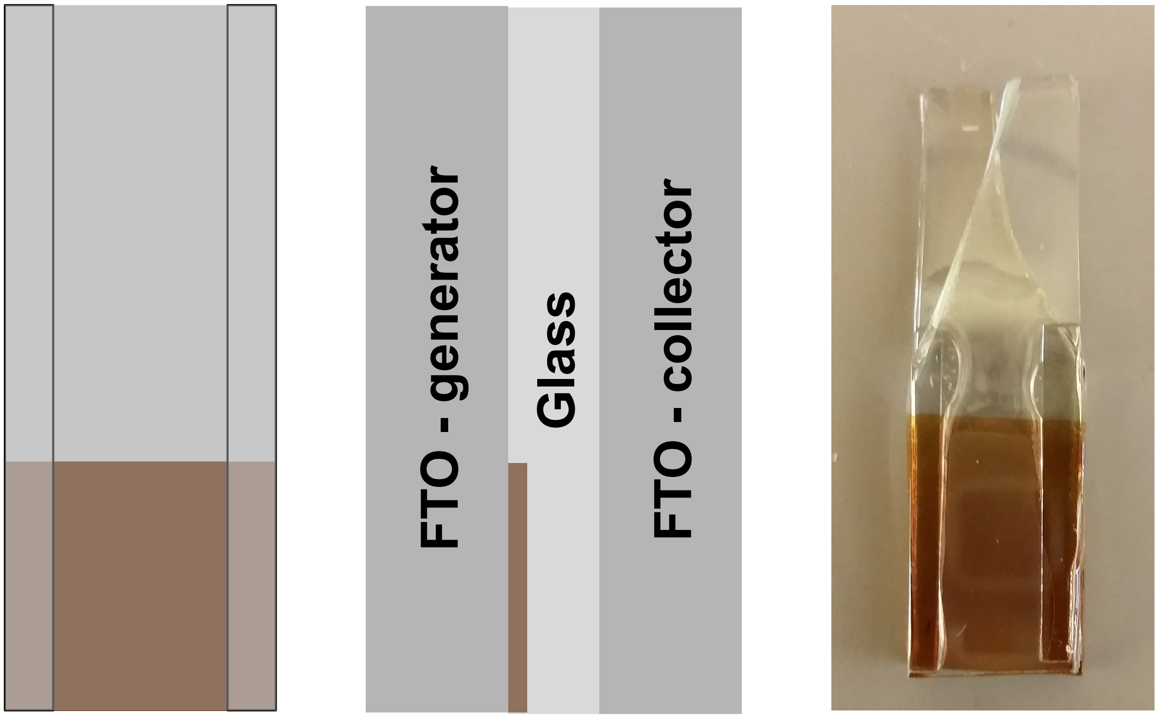

Front View **Side View** **Actual**

Figure S1. Schematic view and photograph of dual working “FTO collector-generator” electrodes.

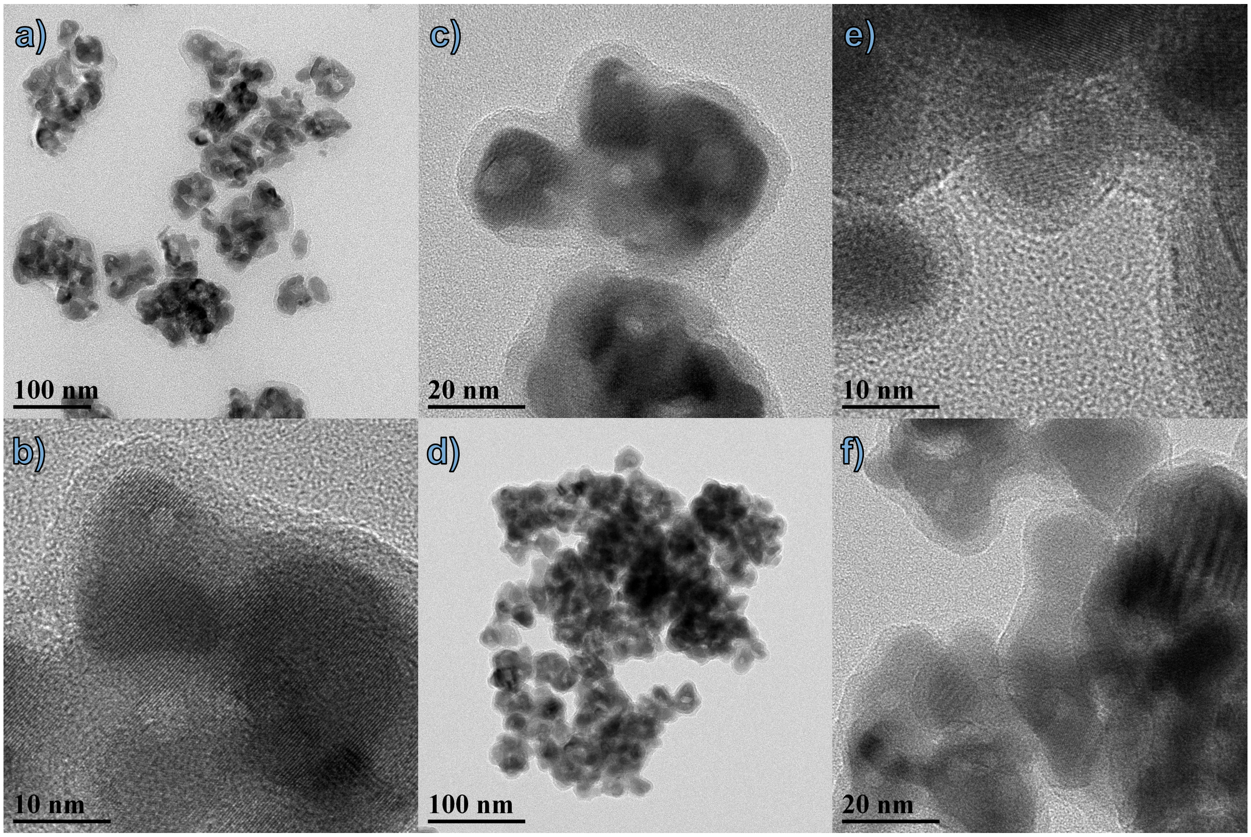

Figure S2. Transmission electron microscopy images of *nano*ITO|-**RuP**$^{2+}$/Al_2O_3 (a, b, c) and *nano*ITO/Al_2O_3 (d, e, f) core/shell structure (core = *nano*ITO|-**RuP**$^{2+}$ or *nano*ITO; shell = Al_2O_3). The Al_2O_3 shell was formed by 20 cycles of $Al(CH_3)_3/H_2O$.

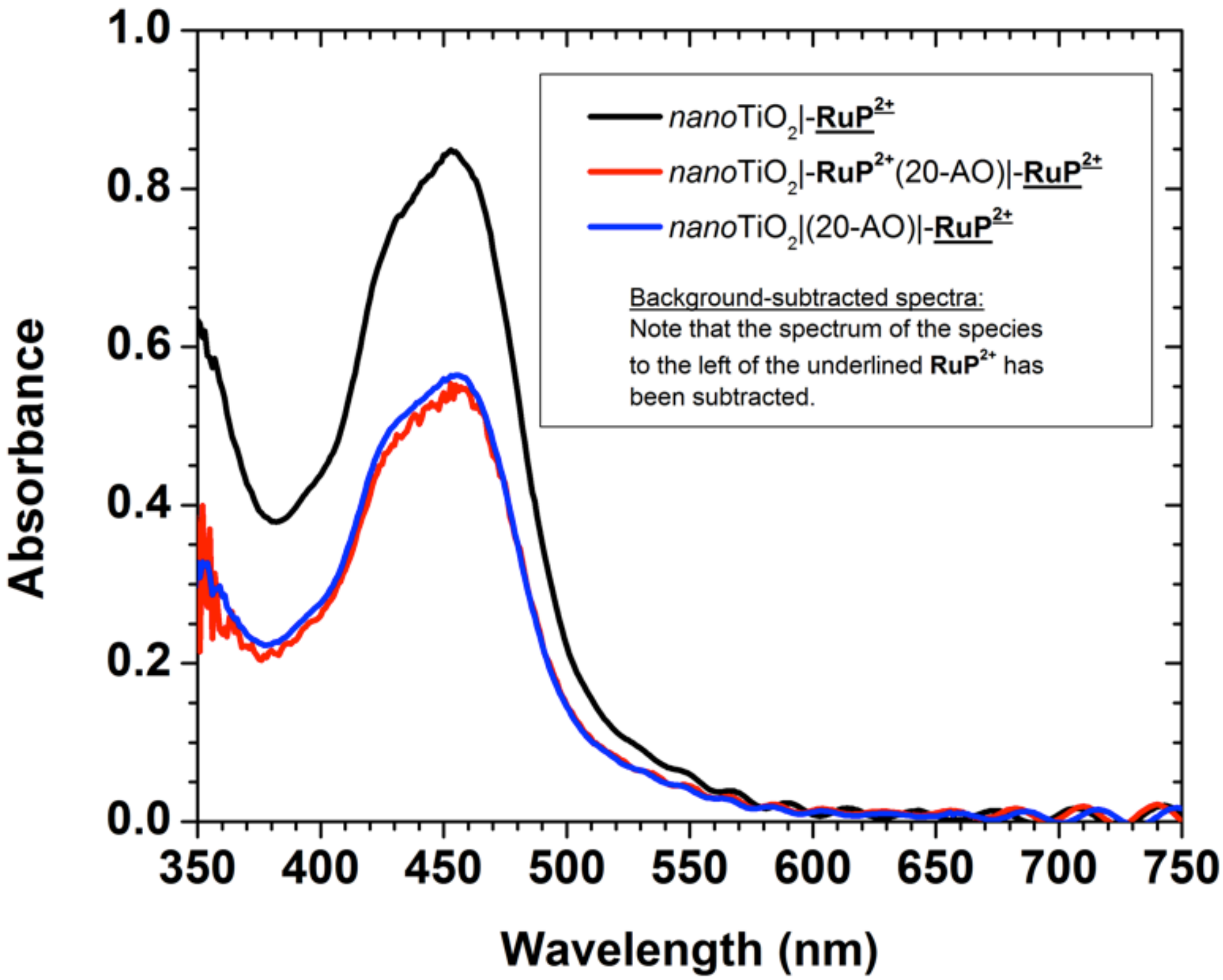

Figure S3. Background-subtracted UV-visible absorption spectra of *nano*TiO_2|-**RuP^{2+}**(20-AO)|-**RuP^{2+}**, *nano*TiO_2|-**RuP^{2+}**, and *nano*TiO_2(20-AO)|-**RuP^{2+}**. Note that background (i.e. the spectrum of all species to the left of the underlined species above) has been subtracted from each spectra as to only present the absorption spectrum of the indicated molecular species.

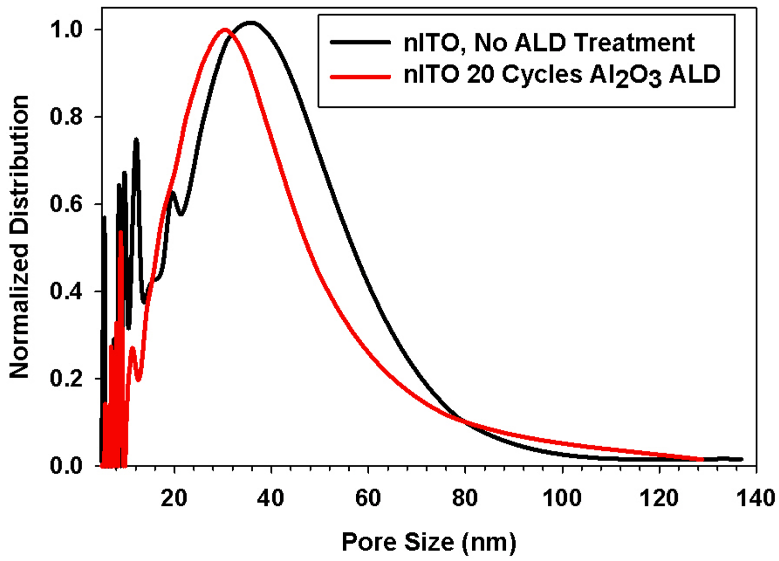

Figure S4. Pore size distribution for *nano*ITO (black trace) and *nano*ITO(20-AO) (red trace).

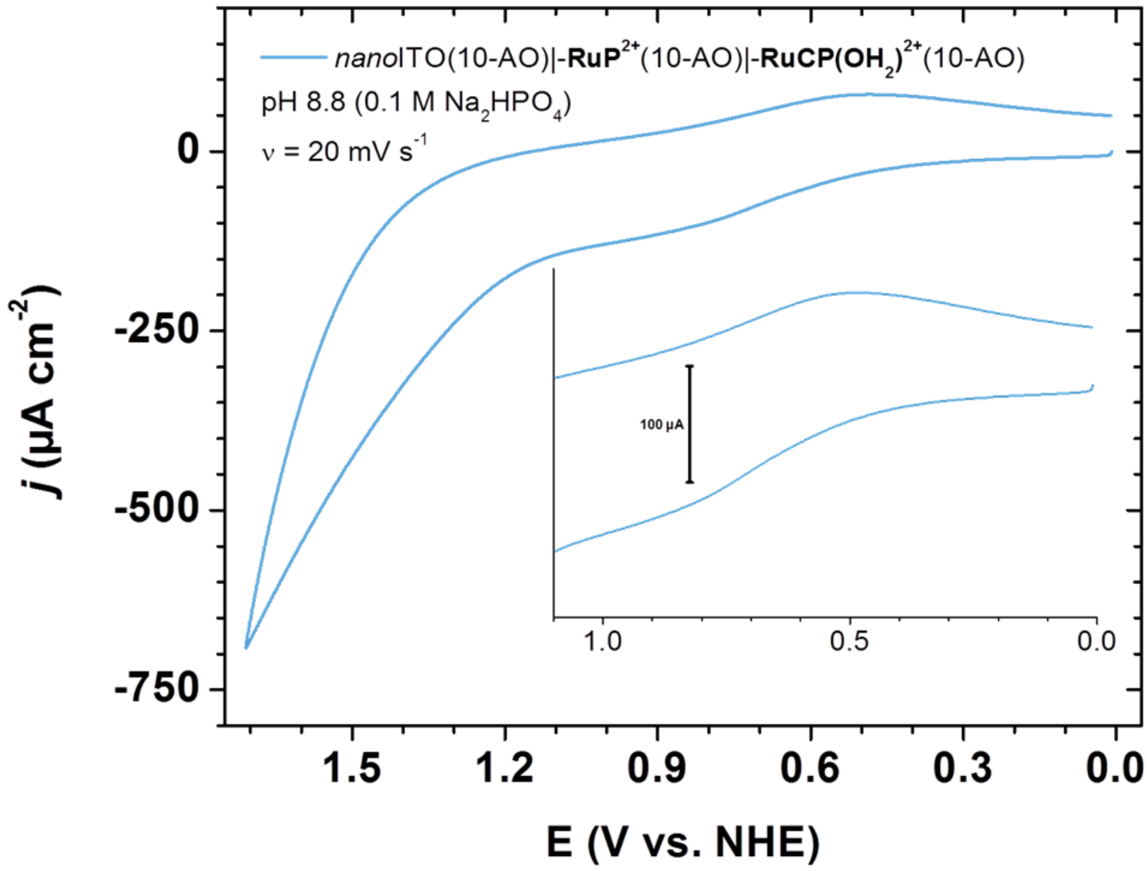

Figure S5. CV scan at pH 8.8 of *nano*ITO(10-AO)|-**RuP**$^{2+}$(10-AO)|-**RuCP(OH$_2$)**$^{2+}$(10-AO) (Conditions: ν = 20 mV s^{-1}; Ag/AgCl, 3 M NaCl reference electrode; Pt-mesh counter electrode)

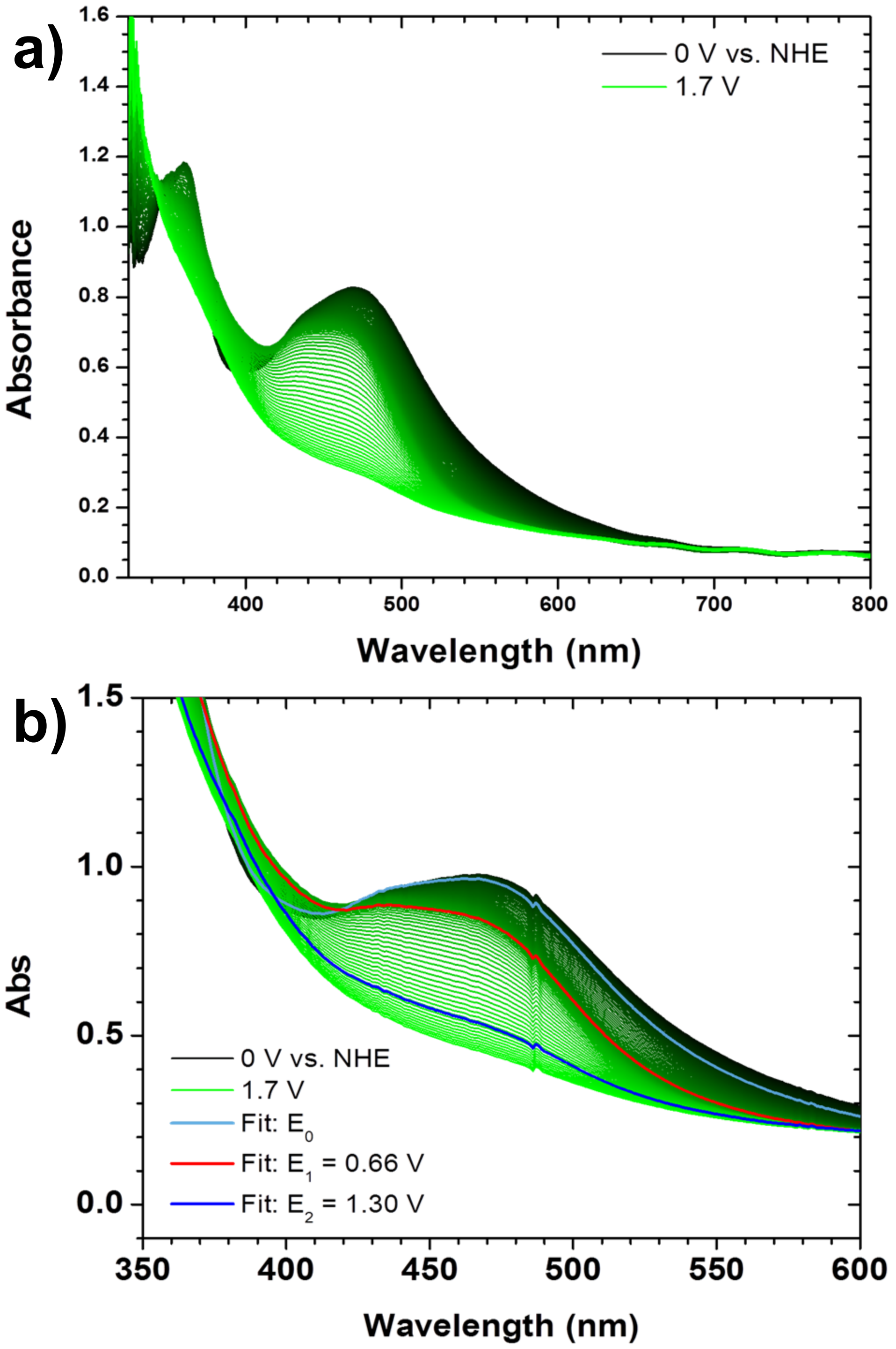

Figure S6. Spectroelectrochemistry for *nano*ITO(10-AO)|-**RuP**$^{2+}$(10-AO)|-**RuCP(OH$_2$)**$^{2+}$(10-AO) with bare *nano*ITO(10-AO) subtracted from each spectrum: a) raw data; b) including fit spectra for ground state (light blue), singly-oxidized catalyst (red), and oxidized chromophore-catalyst (dark blue). (Conditions: 0 to 1.7 V vs. NHE, 0.02-V step, 180-s hold per step; pH 8.8 sodium phosphate dibasic (0.1 M), $NaClO_4$ (0.4 M); Ag/AgCl, 3 M NaCl reference electrode; Pt-mesh counter electrode)

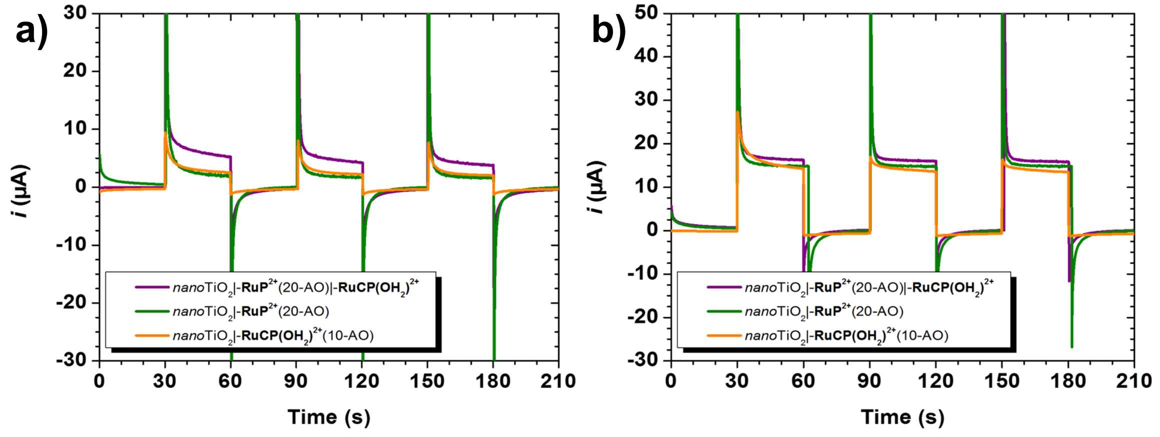

Figure S7. Photoelectrochemical "off-on" traces (~100 mW cm^{-2} illumination, 380-nm long-pass filter) of *nano*TiO_2|-**RuP**$^{2+}$(20-AO)|-**RuCP(OH$_2$)**$^{2+}$ (purple traces), *nano*TiO_2|-**RuP**$^{2+}$(20-AO) (green traces), and *nano*TiO_2|-**RuCP(OH$_2$)**$^{2+}$(10-AO) (orange traces) in pH 4.7 HOAc/NaOAc (0.1 M) buffer a) without; b) with 20 mM hydroquinone added. (Conditions: SCE reference electrode, Pt-mesh counter electrode; solution de-aerated with N_2). NB: The area of each electrode was approximately 1-cm^2.

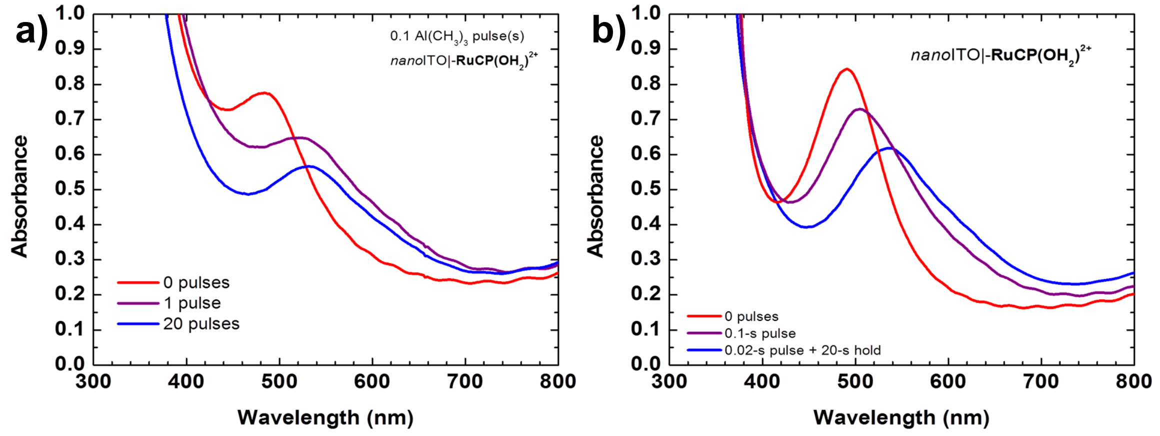

Figure S8. UV-vis absorption spectra of *nano*ITO|-**RuCP(OH$_2$)**$^{2+}$ following a) single pulses of $Al(CH_3)_3$ in the ALD reactor; and b) a single pulse vs. exposing the slide to $Al(CH_3)_3$ for 20 seconds. The red shift is attributed to Ru(II)-OH_2 → Ru(II)-$OAl(OH)_2$.

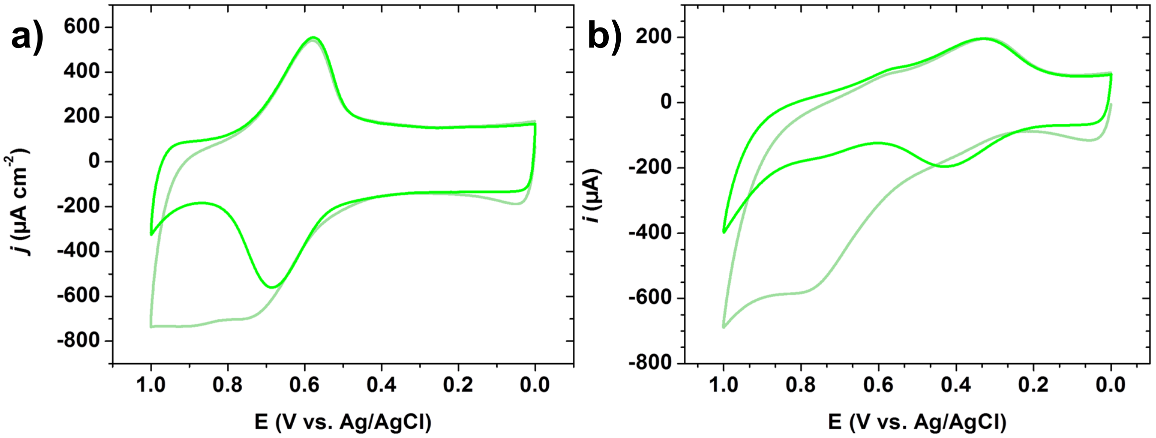

Figure S9. CV scans of *nano*ITO|-**RuCP(OH$_2$)$^{2+}$** + 1 Al(CH$_3$)$_3$ pulse in a) 0.1 M HClO$_4$; and b) 0.1 M sodium phosphate buffer (pH 6.8). In both a) and b), the faded trace is the first scan, while the solid trace is the second scan. The pH-dependent couple is attributed to Ru(II)-OH$_2$, which is regenerated by the second scan in each case.

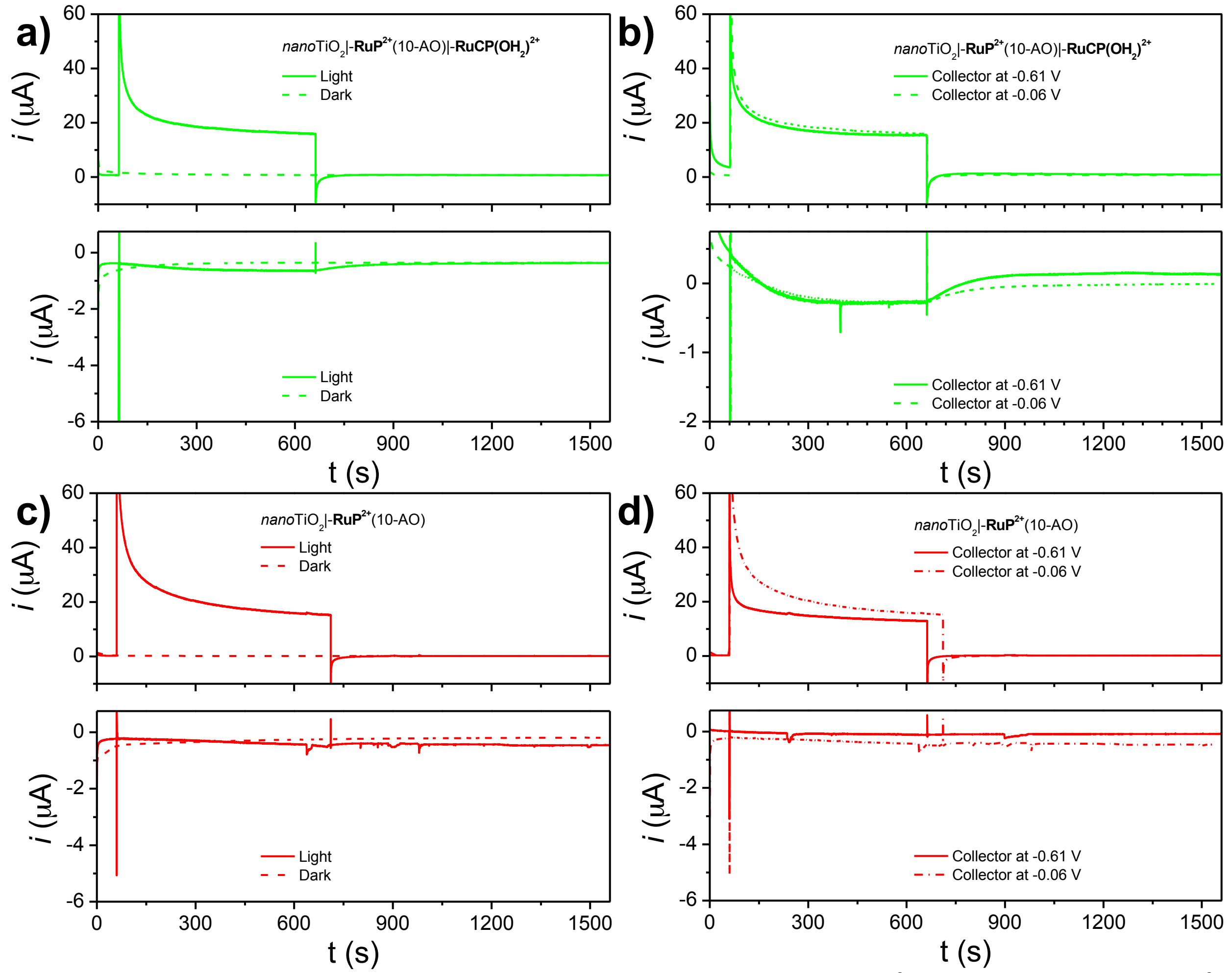

Figure S10. a and c) Photocurrent-time traces for a) *nano*TiO$_2$|-**RuP^{2+}**(10-AO)|-**RuCP(OH$_2$)$^{2+}$** and c) *nano*TiO$_2$|-**RuP^{2+}**(10-AO) at the (top) generator electrode and (bottom) collector electrode

under illumination (solid traces) and in the dark (dashed traces) with E_{gen} = 0.64 V vs. NHE and E_{coll} = -0.61 V vs. NHE. b and d) Photocurrent-time traces for b) *nano*TiO_2|-**RuP**$^{2+}$(10-AO)|-**RuCP(OH$_2$)**$^{2+}$ and d) *nano*TiO_2|-**RuP**$^{2+}$(10-AO) at the (top) generator electrode and (bottom) collector electrode under illumination with E_{gen} = 0.64 V vs. NHE and E_{coll} = -0.61 V vs. NHE (solid traces) or E_{coll} = -0.06 V vs. NHE (dashed traces). (Conditions: ~200 mW cm^{-2} white light illumination; 380-nm long-pass filter; pH 8.8, 0.1 M $H_2PO_4^-/HPO_4^{2-}$; 0.4 M $NaClO_4$; Ref = SCE; Aux = Pt-mesh)

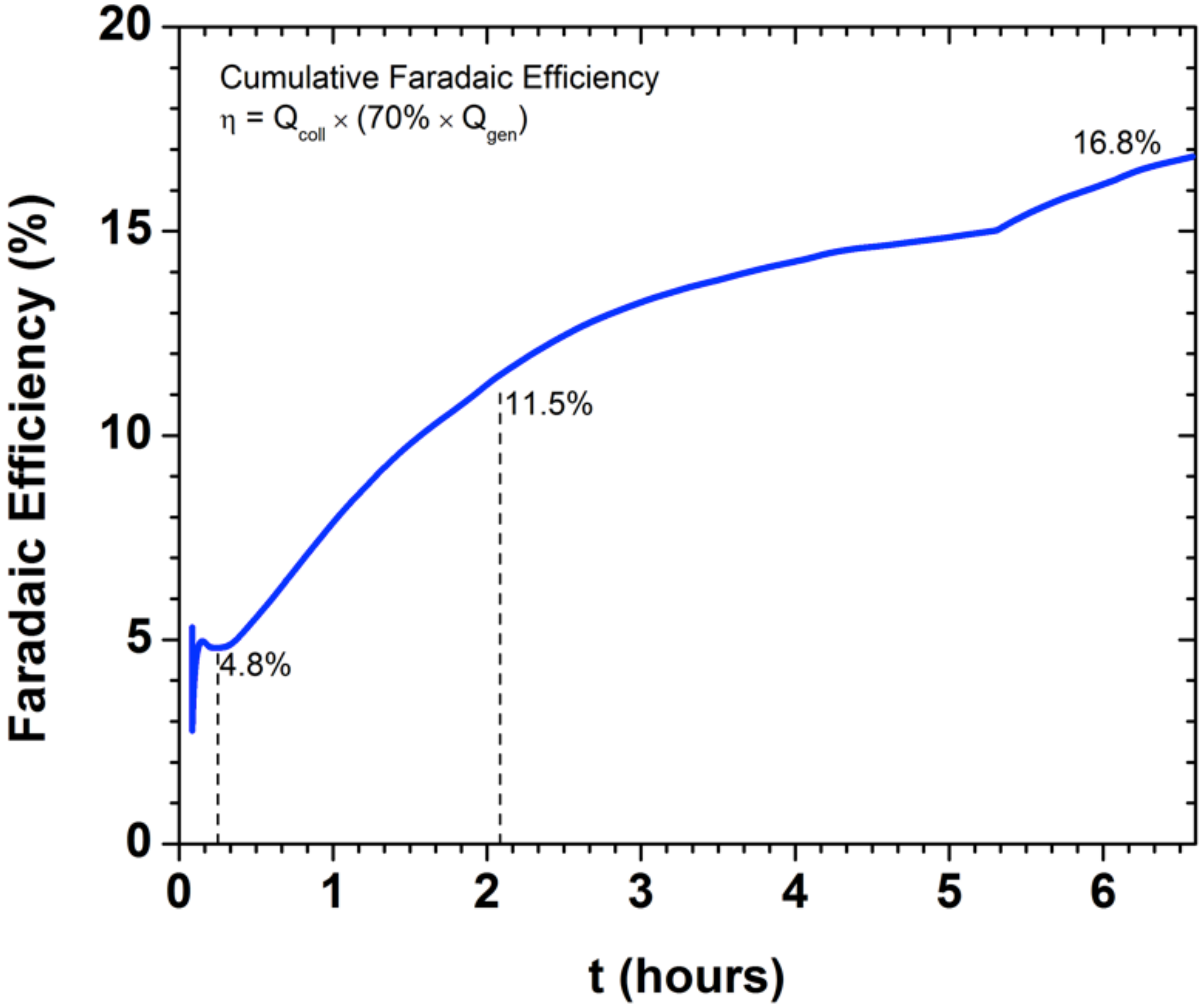

Figure S11. Cumulative Faradaic efficiency vs. Time trace for *nano*TiO_2|-**RuP**$^{2+}$(10-AO)|-**RuCP(OH$_2$)**$^{2+}$(10-AO) under white light illumination. (Conditions: ~200 mW cm^{-2} white light illumination; 400-nm long-pass filter; pH 8.8, 0.1 M $H_2PO_4^-/HPO_4^{2-}$; 0.4 M $NaClO_4$; Ref = SCE; Aux = Pt-mesh; E_{gen} = 0.64 V vs. NHE and E_{coll} = -0.61 V vs. NHE)

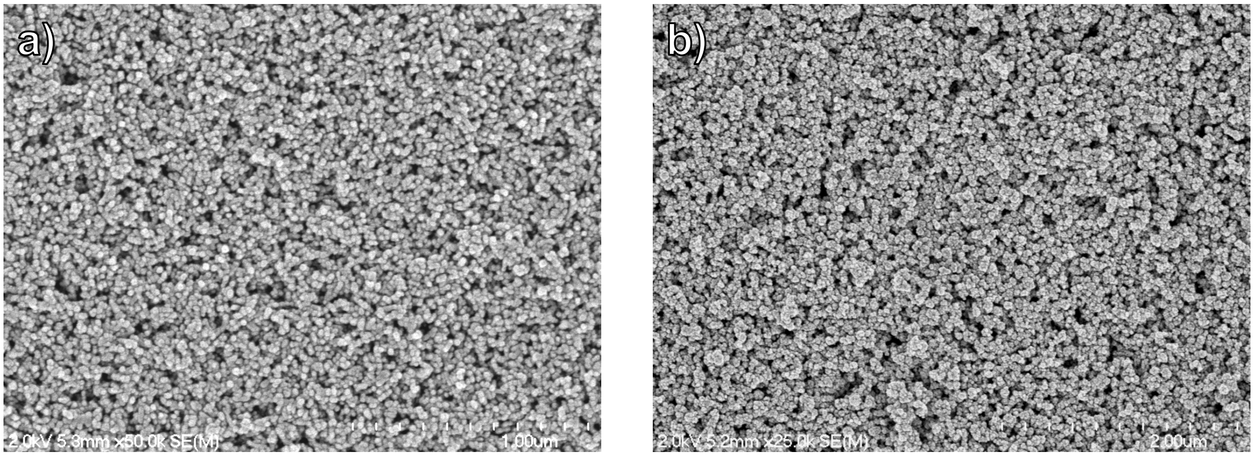

Figure S12. SEM images of (a) *nano*TiO_2 and (b) *nano*ITO.

Additional References:

1. M. R. Norris, J. J. Concepcion, C. R. K. Glasson, Z. Fang, A. M. Lapides, D. L. Ashford, J. L. Templeton and T. J. Meyer, *Inorg. Chem.*, 2013, **52**, 12492-12501.
2. J. J. Concepcion, J. W. Jurss, M. R. Norris, Z. Chen, J. L. Templeton and T. J. Meyer, *Inorg. Chem.*, 2010, **49**, 1277-1279.
3. J. J. Concepcion, R. A. Binstead, L. Alibabaei and T. J. Meyer, *Inorg. Chem.*, 2013, **52**, 10744-10746.
4. J. J. Concepcion, J. W. Jurss, P. G. Hoertz and T. J. Meyer, *Angew. Chem. Int. Ed.*, 2009, **48**, 9473-9476.
